# Enhanced Energy Conversion of Z907-Based Solar Cells by Cucurbit[7]uril Macrocycles

**DOI:** 10.3389/fchem.2019.00561

**Published:** 2019-08-08

**Authors:** Hmoud Al-Dmour, Reem H. Alzard, Hamda Alblooshi, Khaula Alhosani, Shaqra AlMadhoob, Na'il Saleh

**Affiliations:** ^1^Department of Physics, Faculty of Science, Mu'tah University, Mu'tah, Jordan; ^2^Chemistry Department, College of Science, United Arab Emirates University, Al Ain, United Arab Emirates

**Keywords:** solar cell, cucurbit (*n* = 7, 8) uril, metal to ligand charge transfer, charge recombination and separation, electron injection

## Abstract

A dye-sensitized solar cell was constructed on the basis of encapsulating the ruthenium polypyridyl photosensitizer Z907 in the macrocycle cucurbit[7]uril (CB7). The work focuses on the photophysical properties of the new host-guest complexes in acetonitrile and water (volume ratio 1:9) and on the top of nanocrystalline titanium dioxide (TiO_2_) electrode prior to the addition of poly(3-hexylthiophene) polymer and gold electrode. Complexation to CB7 in aqueous solutions has decreased the emission intensity and excited-state lifetime for metal-to-ligand charge transfer (MLCT) state at 650 nm by twofold because of collisional quenching, which opens a non-radiative deactivation channel. Similarly, a twofold decrease in the emission intensity and excited-state lifetime of MLCT at 750 nm on the top of TiO_2_ electrodes was observed with the addition of CB7. Encapsulation of Z907 dye to CB7 host has, also, led to fourfold enhancement in the short circuit current and power conversion efficiency of the final solar cell. The results support the premise that host-guest complexation of CB7 facilitates faster electron injection from Z907 dye into the conduction band of TiO_2_ electrodes.

## Introduction

**Figure 1:**
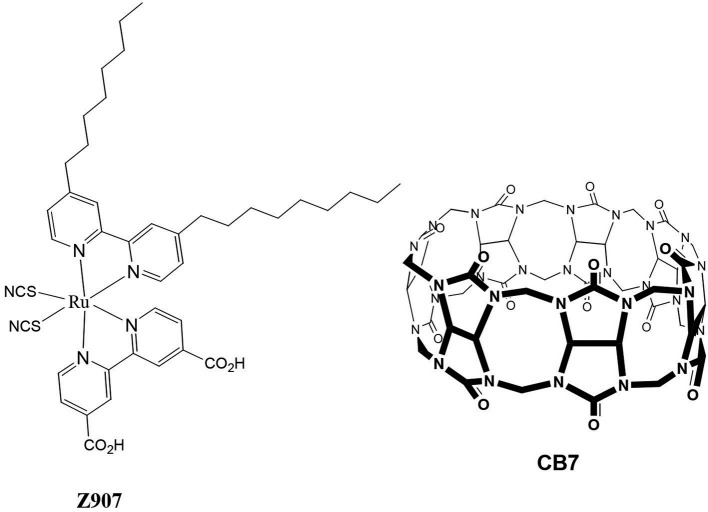
Molecular structures of the proposed dye (Z907) and cucurbit[7]uril (CB7) macrocycle.

Over recent years, several researchers across the globe have geared their efforts toward the enhancement of solar energy conversion, mostly focusing on dye sensitized solar cells (DSSCs) (Robertson, 2006). Organic solar cells have emerged as cheap, robust, and efficient photovoltaic devices, which facilitate the production of high solar energy. They are characterized by a high optical absorption coefficient (usually >10^5^ L.mol^−1^.cm^−1^), low cost of the basic material, a large interface area, compatibility with a flexible substrate, and a good response to high temperature and low light intensities (Robertson, 2006) The simplest configuration of an organic solar cell is to have the organic material placed between two electrodes of different work functions. The system comprises a dye that is bound to the surface of an inorganic semiconductor, such as *cis-*RuLL'(NCS)_2_ (L = 4,4′-dicarboxylic acid-2,2′-bipyridine and L′ = 4,4′-dinonyl-2,2′-bipyridine) (Z907) dye (Wang et al., 2003a,b,c) and nanocrystalline titanium dioxide (TiO_2_) semiconductor, which were selected for the present study (Figure 1). Excitation of Z907 leads to the injection of electrons from the excited dye into the conduction band of the TiO_2_. This injection was not very effective under specific conditions (Wang et al., 2005), which motivated us to conduct the present study (see below). The dye-sensitized nanocrystalline TiO_2_ provides a large surface area to which the dye could be adsorbed. This is crucial for efficient light harvesting. The porous TiO_2_ layer is then interpenetrated by a hole-transport material (HTM), which is a solid-state or quasi-solid-state (gel) material. In the present work poly(3-hexylthiophene) polymer (P3HT) (Al-Dmour and Taylor, 2011) was selected as the HTM encouraged by a previous report, in which P3HT electrolyte was also used with Z907 to improve cell performance (Schmidt-Mende et al., 2005). The ground state of Z907 was regenerated through reduction by the P3HT to give the required charge separation. Charges were migrated and collected at the transparent conducting electrode, SnO_2_ and Au. The efficiencies of the final constructed solar cell were measured in terms of the overall efficiency of conversion of solar-to-electrical energy of the cell (η) and the incident photon-to-current efficiency (IPCE) (Al-Dmour et al., 2007).

Several researchers have attempted to improve the efficiency of organic solar cells by utilizing the supramolecular approach (Haque et al., 2004; Handa et al., 2007; Choi et al., 2009; Wu et al., 2009). Supramolecular chemistry is focused on intermolecular non-covalent interactions between molecules that lead to formation of macromolecular assemblies (Steed and Gale, 2012). The structures and properties of the resulted assemblies differ (often better) from those of their individual components because of these intermolecular interactions (electrostatic, hydrogen bonding, van der Waals, and donor-acceptor). When guest molecules are non-covalently encapsulated inside macrocyclic containers, a modification of their chemical and physicochemical properties always results due to the altered microenvironment as well as the confinement and isolation of the guest (Koner and Nau, 2007). The encapsulation essentially reduces the tendency of the guest to undergo aggregation or unspecific adsorption. Further, the complexation may enhance redox reversibility, electroluminescent efficiency (Freitag and Galoppini, 2010), thermal stability and photochemical stability (Zhang et al., 2012).

Haque et al. (2004) studied an azobenzene dye, which was encapsulated within a cyclodextrin (CD) molecule, then attached to TiO_2_. The CD comprises a hydrophilic outer layer, which is suitable for adsorption to the TiO_2_ surface, and a hydrophobic inner surface. The spatial separation of the dye from the TiO_2_ offered a potential gain in cell efficiency. In particular, the retardation of charge recombination in Z907-based solar cells by the adsorption of CD macrocycles was also reported by the same research group (Handa et al., 2007). Moreover, Choi et al. (2009) encapsulated other small organic dyes inside CD cavities, which provided an overall conversion efficiency of 7.4%. Also, they found the new CD-based device to have excellent thermal and photochemical stability. They attributed the high efficiency and excellent stability to the encapsulation of their dye inside the CD cavity.

However, recent reports (Pagba et al., 2004; Mohanan et al., 2019) on the effective electron injection from CB host-guest complexes into semiconductors' nanoparticles (including TiO_2_) have motivated us to select cucurbiturils (CBs) (Lee et al., 2003; Masson et al., 2012) over CD molecular containers in the present investigation. In addition, stable adsorption of CB molecular containers to TiO_2_ was established (Freitag and Galoppini, 2010) on the basis of the unique tendency of CB to strongly bind cationic guest molecules through ion-dipole interactions between the carbonyl portals of CB and the metal ions or metal oxides (Lee et al., 2003; Masson et al., 2012). Indeed, CB7 encapsulated-rhodamine B dye (RhB) was utilized this year to enhance an electron transfer from the dye to TiO_2_, thus facilitating faster mineralization of the dyes under solar irradiation (Mohanan et al., 2019). Wu et al. (2009) also reported in an early study the effects of encapsulation of a dye molecule by CB7 on the charge-recombination dynamics. Comparison of the transient emission behaviors of the free and CB7-bound dye revealed that encapsulation inside CB7 suppresses the loss mechanism. In light of the above, we sought in this work to examine the effects of the addition of cucurbit[7]uril(CB7) macromolecules (Figure 1) on the efficiency of the final Z907-based solar cells.

## Results and Discussion

### Interactions of Z907 With CB7 in Aqueous Solution and Solid State

There are two bands known for Z907 dye in neat solvents: the ligand-to-ligand charge transfer (LLCT) and metal-to-ligand charge transfer (MLCT) (Wang et al., 2003a,b,c). See Figure S1 for Z907 in acetonitrile and water (volume ratio 1:9). The excitation spectra display two bands at 290 and 480 nm, when the emission wavelength was set at 550 and 650 nm, respectively (Figure S2a). Moreover, when exciting the sample at 350 and 400 nm, two different emission bands were observed at 430 and 650 nm, respectively (Figure S2b). The results confirm that the LLCT and MLCT absorb at 290 and 478 nm and emit at 430 and 650 nm, respectively. The titration of Z907 by CB7 in this aqueous solution using absorption spectroscopic measurements has demonstrated weak interactions between the dye and host in the ground state. The mode of interaction can be revealed by NMR titration. However, the data in Figure S3 reveal no spectral changes in the DMSO-d_6_ solvent. The organic solvent may have restricted the host-guest complexation process. Unfortunately, the very low solubility of Z907 in D_2_O has prevented us from obtaining useful NMR data. However, we have measured the FTIR spectra for Z907, CB7 and a solid host-guest complex from the two reactants. The results in Figure S4 in the Supporting Information revealed a significant change in the stretching IR bands owing to the complexation process. In particular, a shift for C-H stretching band from 1,900 cm^−1^ to 1,800 cm^−1^ indicates the engulfing of the alkyl chain by CB7, which agrees with a similar binding mode in other CB7-complexed ruthenium dyes (Sun et al., 2007).

### Spectrophysics of Z907 in Solution

**Figure 2:**
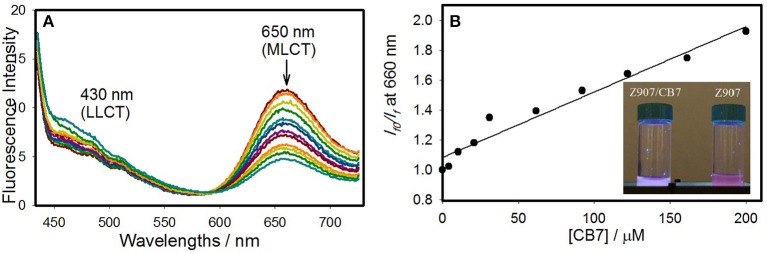
Fluorescence changes **(A)** as a function of CB7 (0–40 equivalents) for Z907 (30 μM) in acetonitrile and water (volume ratio 1:9) at pH 7 and room temperature upon excitation at 375 nm. And the nonlinear fitting **(B)** according to a 1:1 binding model, see the Experimental Section.

The interactions of CB7 with Z907 in the excited state was demonstrated in solution. Figure 2 illustrates a comparison of the steady-state photoluminescence (PL) for the free and CB7-complexed Z907 (30 μM) in acetonitrile and water (volume ratio 1:9) at pH 7. The intensity of the emission band at 650 nm (MLCT state) has diminished upon complexation to CB7 by 10-fold. The data were used to construct a Stern-Volmer plot (inset in Figure 2), which supports the diffusion-controlled quenching process by CB7 quenchers (height ~ 9.1 A^o^) (Valeur, 2002). The measured and calculated bimolecular rate constant (*k*_q_) were 1.05 × 10^10^ M^−1^s^−1^ and 7.83 × 10^9^ M^−1^s^−1^. The proximity of the two values confirm the small contribution of the static component (see FTIR results above) in the overall dynamic quenching. The quenching rate constant (*k*_*q*_) was calculated from the known Smoluchowski (Equation 1) and Stokes-Einstein (Equation 2) relations, assuming pure diffusion-limited process (*k*_diff_).

(1) $$\begin{array}{r} k_{q}=k_{diff}=4\pi NR_{q}D \end{array}$$

where *R*_q_ is the distance of the closet approach (in m), *D* is the mutual diffusion coefficient (in m^2^ s^−1^), and *N* is equal to 1000 *N*_*a*_ (*N*_*a*_ being the Avogadro's number). The mutual diffusion coefficient is the sum of the translational diffusion coefficients of the fluorophore (Z907) and the quencher (CB7).

*D*_M_ and *D*_Q_, respectively, and can be expressed by the Stokes-Einstein relation (Equation 2)

(2) $$\begin{array}{r} D=D_{M}+D_{Q}=\frac{\kappa T}{6\pi \eta }\left(\frac{1}{R_{M}}+\frac{1}{R_{Q}}\right) \end{array}$$

where κ is Boltzman's constant (1.381 × 10^−23^ kgm^2^s^−2^K^−1^), η is the viscosity of the medium in kg m^−1^s^−1^ (for water η equals 8.95 × 10^−4^ at 298 K) (Valeur, 2002) and *R*_M_ and *R*_Q_ are the radii of the hydrated fluorophore (Z907) (Pan et al., 2011) and hydrated quencher (CB7) (Lee et al., 2003) in m, respectively. The values substituted were 5.6 × 10^−10^ and 9.1 × 10^−10^ m, respectively.

**Figure 3:**
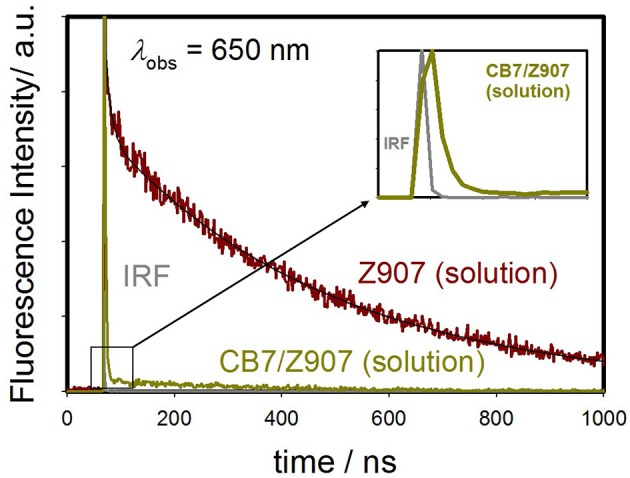
Emission decays monitored at 650 nm for Z907 (30 μM) in acetonitrile and water (volume ratio 1:9) (pH 7, room temperature) and with CB7 (10 equivalents) upon excitation at 375 nm. The *inset* expands the region in 10 ns range to unfold the IRF decay profile.

In addition to steady-state measurements, the time-resolved photoluminescence (TRPL) spectra were also measured to better comprehend the type of fluorescence quenching in solution and the nature of CB7-induced supramolecular effects on the photophysical properties of Z907. The excited-state lifetimes for the free and CB7-bound Z907 in acetonitrile and water (volume ratio 1:9) at pH 7 are listed in Table 1. The emission decays for the free and CB7-bound Z907 were monitored at 430 (Figure S5) and 650 nm (Figure 3) for each sample when excited at 375 nm. All emission traces were fitted to bi- or tri-exponential model functions after being convoluted with IRF of ~30 ps. The emission spectra were collected for the samples under nitrogen several times with an estimated error for the measured fluorescence lifetime of about 4%.

**Table 1:** Excited-state lifetime constants observed at 430 and 650 nm for Z907 (30 μM) in acetonitrile and water (volume ratio 1:9) and inside CB7 cavity (10 equivalents).

**pH**	**λ_obs_ (nm)**	**τ_1_ (ns)**	***f*_1_%**	**τ_2_ (ns)**	***f*_2_%**	**τ_3_ (ns)**	***f*_3_%**	**τ_4_ / ns**	***f*_4_%**	**τ_average_ (ns)**	**Chi Square**
7.0 (Free)	430	35	55	1.56	20	7.36	25			2.17	1.034
	650					9.79	1	418	99	414	1.051
7.0 (Complex)	430	43	53	1.60	21	8.88	26			2.52	1.035
	650			1.82	36			269	64	173	1.202

*Time resolution was ~30 ps and excitation wavelength was 375 nm*.

The Z907 exhibits four lifetime components at 430 nm (Figure S5). All species originated from a separate ground-state and can be attributed to LLCT and MLCT bands (see above). While the long-lived species at ~400 ns appear to be associated with MLCT, further experiments are needed to explore the origin of the three other transient components, which belong to LLCT (~0.4, 1.6, and 7.4 ns). Noticeably, upon the addition of CB7, the emission decay at 650 nm was most significantly affected, confirming the ability of CB7 to intervene with the MLCT process in Z907. From the twofold decrease in this excited state lifetime at 650 nm (from ~400 to 270 ns in Table 1), it is plausible to associate such lifetime suppression to a collisional quenching via a metal-to-CB7 charge transfer. Such interaction has created a non-radiative deactivation channel for MLCT emission. The results also explain the enhancement in solar cell efficiency with the addition of CB7, see below.

### Spectrophysics of Z907 on the Top of Electrodes

**Figure 4:**
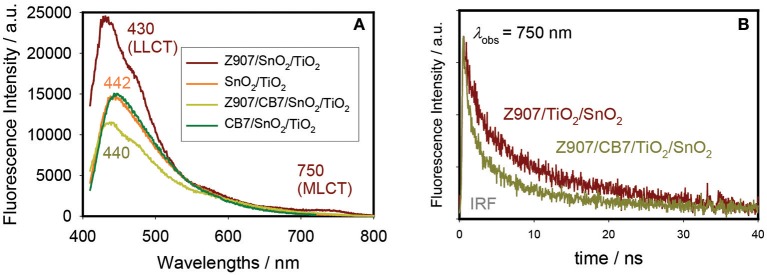
Steady-state photoluminescence (PL) spectra **(A)** and emission decays at 750 nm **(B)** of Z907/TiO_2_/SnO_2_ and Z907/CB7/TiO_2_/SnO_2_ electrodes. PL for CB7-coated and uncoated TiO_2_/SnO_2_ electrodes are also shown in A for comparison.

The PL spectra for Z907/TiO_2_/SnO_2_ and Z907/CB7/TiO_2_/SnO_2_ electrodes along with the spectra for CB7-coated and uncoated TiO_2_/SnO_2_ electrodes are shown in Figure 4. The Z907-coated electrodes show the expected two bands due to LLCT and MLCT at 430 and 750 nm, respectively. As a control experiment, CB7 alone did not affect the emission profile of the uncoated electrodes. However, for the complex Z907/CB7-coated electrodes, the emission profiles of the uncoated electrodes were restored (Figure 4A) when compared to that of Z907/TiO_2_/SnO_2_ electrode. In addition, the average excited-state lifetime of the 3 components at 440 nm (LLCT band) was restored to that of the uncoated electrodes upon the addition of CB7 to Z907-coated electrodes (from ~3 ns to ~1 ns, Table 2 and Figure S6). For CB7-coated electrodes, the excited-state lifetime for the LLCT band was enhanced by twofold when compared to that of the uncoated electrodes (from ~1 ns to ~2 ns, Table 2 and Figure S6). At 750 nm (MLCT band) the average excited-state lifetime has decreased from ~18 ns to 10 ns (Table 2 and Figure 4B), confirming that CB7 enhances the electron transfer (sensitization) from the dye into the conduction band of TiO_2_ (see below) (Pagba et al., 2004; Hara et al., 2005; Zhang et al., 2012; Mohanan et al., 2019).

**Table 2:** Excited-state lifetime constants observed at 440 and 650 nm for different Z907-based solar cell electrodes.

**Electrode type**	**λ_obs_ (nm)**	**τ_1_ (ns)**	***f_1_%***	**τ_2_ (ns)**	***f_2_%***	**τ_3_ (ns)**	***f_3_%***	***Chi Square***	**τ_average_ (ns)**
SnO_2_/ TiO_2_	440	0.10	24	0.58	30	2.45	46	1.256	1.33
SnO_2_/ TiO_2_/ CB7	440	0.24	16	0.97	37	3.29	47	1.026	1.94
SnO_2_/ TiO_2_/ Z907	440	0.03	11	0.62	28	4.58	61	1.267	2.96
	750	0.29	2	3.04	14	20.71	84	1.121	17.86
SnO_2_/ TiO_2_/ CB7/ Z907	440	0.04	20	0.30	26	1.37	54	1.022	0.82
	750	0.39	9	2.91	26	14.49	65	1.141	10.17

*Time resolution was ~30 ps and excitation wavelength was 375 nm*.

### Measurements of Solar Cell Efficiency With and Without CB7

**Figure 5:**
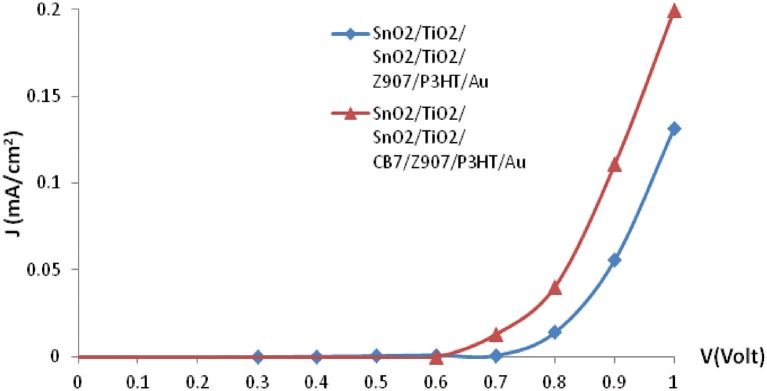
Current-density (J-V) characteristics of the bulk-heterojunction solar cell in dark.

The photovoltaic properties of the Z907-based solar cells by CB7 macrocycles were characterized by measuring the current density-voltage (J-V) curves in the dark and under white light illumination though the SnO_2_:Fn side. The characteristic parameters of the Z907 solar cells were obtained by plotting J-V curves in linear scales. Figure 5 shows the forward bias's current density–voltage (J-V) characteristic under darkness. The onset voltage for conduction in forward bias decreases from 0.8 V in the device without CB7 to 0.7 V for the device consisting of CB7-absorbed TiO_2_ particles. Also, addition of CB7 enhances significantly the current density for conduction in forward bias.

**Figure 6:**
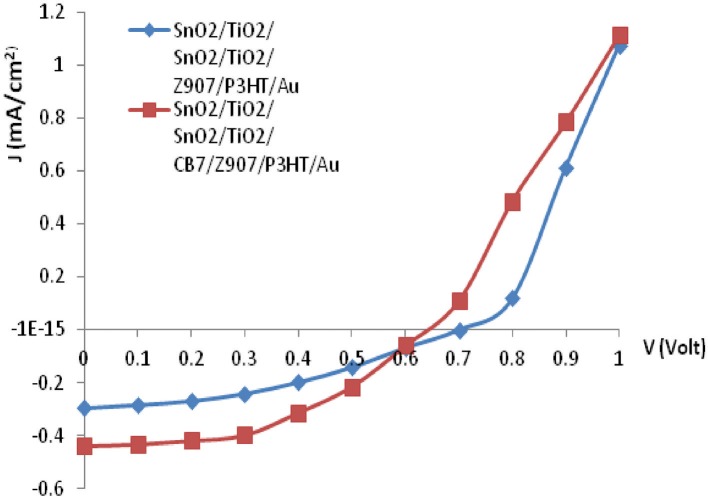
Current-density (J-V) characteristics of the bulk-heterojunction solar cell under illumination (AM 1.5, 100 mW/cm^2^).

The J-V characteristic for the device under AM 1.5 illumination with an overall intensity of 100 mW/cm^2^ is shown in Figure 6. The short circuit density, Jsc, of the CB7-containing device has increased from 0.29 mA/cm^2^ to 0.43 mA/cm^2^, whereas its open circuit voltage, Voc, has reduced from 0.7 to 0.65 V in comparison with the device without CB7. Moreover, addition of CB7 has increased the power conversion efficiency of the final solar cell by more than 50% using the equation reported in our previous work (Na'il Saleh et al., 2015) (from 0.08 to 0.12%). This improvement in short circuit current and power conversion efficiency of our devices agree with previous reports (Haque et al., 2004; Handa et al., 2007; Choi et al., 2009). On the contrary to our previous work, the Z907/CB7-based solar cells show better performance than the reported DSSC device, which was based on the host-guest encapsulation of 5-[4-diphenylamino)phenyl]thiophene-2-cyanoacrylic acid (L1) inside β-CD hosts (Na'il Saleh et al., 2015).

**Figure 7:**
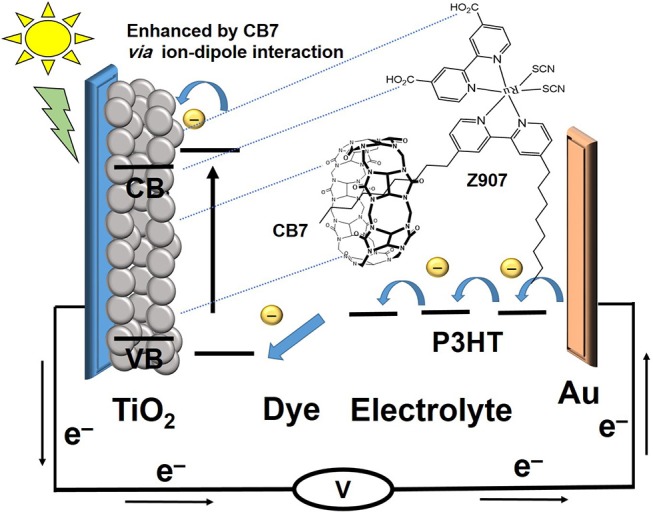
Schematic representation of the CB7-assited transfer from Z907 dye to TiO_2_ semiconductor through ion-dipole interactions between the carbonyl portals of CB7 and the positive surface.

**Figure 8:**
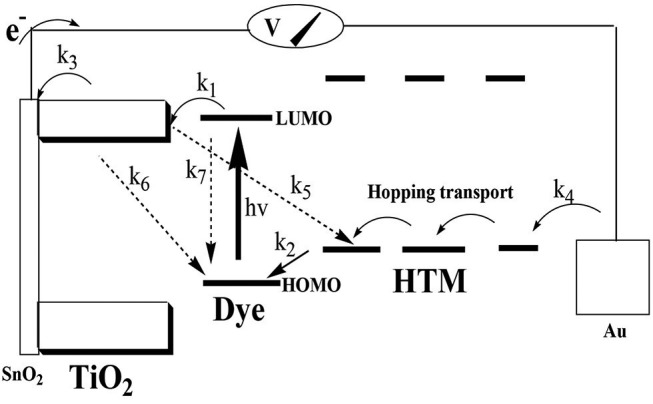
Energy level scheme of the proposed DSSC. Photoinduced electron transfer takes place from the photoexcited dye into the TiO_2_ conduction band. The charge recombination back to the dye must be suppressed. The loss mechanism is represented by the three dashed arrows: *k*_5_ = charge recombination with the hole-transport material (HTM; dark current), *k*_6_ = charge recombination with the oxidized dye, and *k*_7_ = decay of the excited state of the dye. Instead, the current is directed through the circuit to the counter electrode and the HTM that brings the electrons back via hopping transport. The arrows that represent processes required for photovoltaic function are: *k*_1_ = charge injection, *k*_2_ = dye regeneration, *k*_3_ = charge collection at the conducting glass electrode, and *k*_4_ = charge collection at the Au electrode.

The previously reported (Wang et al., 2003a,b,c) photophysical properties of Z907 indicate that the charge-transfer transitions place the excited electron on the carboxylate group, which is directly attached to the TiO_2_ (Galoppini, 2004). The stronger adsorption of the CB7-encapsulated dye to the positively charged TiO_2_ because of the ion-dipole interactions of the carbonyl portals of CB7 must have brought the dye closer to the surface (Figure 7), thus generating faster electron injection from the dye into the semiconductor (large *k*_1_ in Figure 8) (Mohanan et al., 2019). Recall that the COOH group of the dye is free to bind the surface while the dye is encapsulated by CB7 (Figure 7).

In their study of the adsorption of CB7-encaposualted (RhB) dye on TiO_2_, the authors concluded that the negatively charged carbonyl portals hinders recombination of electron-hole pairs (Mohanan et al., 2019). By analogy to their explanation, the spatial separation by CB7 of the positive charge density on Z907 dye and the injected electrons into TiO_2_ has probably retarded the rate of charge recombination between the injected electrons and the dye cation, which is a key loss mechanism (*k*_6_) (Hirata et al., 2004; Haque et al., 2005; Ciofini et al., 2012; Mohanan et al., 2019). It is also plausible to assume that CB7 has prevented the interaction between HTM and the TiO_2_ surface, thus minimizing *k*_5_ loss (Zakeeruddin et al., 2002), promoting consecutive electron-transfer processes between the dye and HTM, which should lead to faster regeneration kinetics (*k*_2_) of the dye (Clifford et al., 2004). Overall, the use of CB7 can replace other electrolytes for enhancing Z907-based solar cell efficiency (Mori et al., 2007; Bai et al., 2008; Cao et al., 2008; Yang et al., 2009; Lee et al., 2014; Wu et al., 2015).

## Experimental

### Reagents and Sample Preparation

Z907, CB7, and acetonitrile (purity > 99.9%) were purchased from Sigma-Aldrich (St. Louise, MO, USA) and used as received. The concentrations of CB7 were calculated on the basis that the host contains 20% water, as notified by the supplier. Millipore water was used (conductivity < 0.05 μS). The Z907/CB7 complex was prepared in the solid state by the grinding method. The initial reactants were mixed in a 1:1 molar ratio, then grounded for about 20 min before the final solid was washed by acetone, then dried off.

### Instrument

Proton-NMR spectra were measured in DMSO-d_6_ using NMR 400 MHz spectrometer (Varian, Palo Alto, CA, USA) with a reference in ppm to a TMS standard. The solid complex was analyzed by FTIR spectrometer (Agilent Technologies Cary 600 Series FTIR). The spectra were recorded in the range of 4,000–400 cm^−1^, averaging 512 scans at a resolution of 2 cm^−1^. The UV–vis absorption spectra were measured on a Cary-300 instrument (Agilent, Santa Clara, CA, USA). Fluorescence spectra in solution were measured using a Cary-Eclipse instrument (Agilent, Santa Clara, CA, USA) with slit widths of 2.5 nm and 5 nm for the excitation monochromator and emission monochromator in all experiments. The photoluminescence (PL) and excited-state lifetimes were collected for both free and CB7-complexed dyes in solution and on the top of electrodes under similar conditions of slit widths by time-correlated single-photon counting (TCSPC) on LifeSpec II spectrometer (Edinburgh Inc., Edinburgh, UK); and by using EPL-375 picosecond diode laser (wavelength of 375 nm, repetition rate of 20 MHz, and instrument function of ~250 ps) for excitation. The re-convoluted lifetimes from the measurements and instrument function cannot be < ~30 ps (one-tenth the instrument function). The time-resolved photoluminescence (TRPL) with intensity of ~1,000 counts/s (in solid) and ~10,000 counts/s (in solution) were collected by a red-sensitive high speed PMT detector (H5773-04, Hamamatsu, Japan). A long pass filter at 420 nm was placed between the sample holder and the emission monochromator during the measurements of the TRPL spectra for electrodes. The data were analyzed by the iterative reconvolution method using the instrument's software that utilizes the Levenberg–Marquardt algorithm to minimize χ^2^. The fluorescence decay was analyzed in terms of the multiexponential model

(3) $$\begin{array}{r} I(t)=\sum_{i}^{}\alpha_{i}\;exp(-t/\tau_{i}) \end{array}$$

where τ_*i*_ are the lifetimes with amplitudes α_*i*_ and ϒ∑_*i*_ = 1.0. The contribution of each component to the steady-state intensity is given by

(4) $$\begin{array}{r} f_{i}=\frac{\alpha_{i}\tau_{i}}{\sum_{j}^{}\alpha_{j}\tau_{j}} \end{array}$$

where the sum in the denominator is over all the decay times and amplitudes. The mean decay time (average lifetime) is given by:

(5) $$\begin{array}{r} \tau =\sum_{i}^{}f_{i}\tau_{i} \end{array}$$

### Solar-Cell Constructions and Testing

The devices studied here were fabricated on fluorine-doped, tin oxide (SnO_2_:Fn) electrodes, pre-coated with a thin, dense layer of TiO_2_. A TiO_2_ sol-gel (Ti-Nanoxide T) was then spread over the substrates using a doctor blade and cured to form the anatase phase following the previously published procedure (Al-Dmour and Taylor, 2011). Typically, the resulting porous, TiO_2_ layer was ~2 μm in thickness. Z907/CB7/SnO_2_/TiO_2_ film was prepared through immersing the SnO_2_/TiO_2_ electrode, first, in the solution of CB7 (0.8 mM in water), then in the dye solution (0.3 mM in ethanol) (Lee et al., 2003). A drop of poly(3-hexylthiophene) polymer (P3HT) in chloroform (15 mg/mL) was suffused into this layer for several seconds prior to spin coating at 1,000 rpm. The devices were completed by evaporating an array of 3 mm^2^, circular gold (Au) electrode onto the P3HT.

### Electrical Characterization

A Keithley Model 237 High-Voltage Source-Measure Unit was used for measuring the current voltage (I-V) in dark and light. A full Spectrum Solar Simulator (150 W Xenon Lamp) was used to obtain the optical response of the solar cells. A mask with identical holes (size and distribution of holes are fixed) was placed against the glass substrate to limit the light to the region defined by the gold top electrode. A light intensity meter was used to measure the light intensity equivalent to AM 1.5 radiation from a Xenon lamp using a reference solar cell.

## Conclusions

The results in the present work support the premise that CB7 host molecule (having negatively charged carbonyl portals) brings the dye (represented here by Z907) closer to the positive surface (represented here by TiO_2_) through ion-dipole interactions between CB7 and the surface. Overall, encapsulation inside the CB7 cavity of the solar cell dye is enough to induce faster electron injection into the conduction band of the semiconductor, thus enhancing the device power conversion by a factor of 4 (the results in the present study).

## Data Availability

The raw data supporting the conclusions of this manuscript will be made available by the authors, without undue reservation, to any qualified researcher.

## Author Contributions

HA-D fabricated the solar cell and measured its efficiency. RA, HA, KA, and SA collected the PL and TRPL data. NS designed the work and wrote the manuscript.

### Conflict of Interest Statement

The authors declare that the research was conducted in the absence of any commercial or financial relationships that could be construed as a potential conflict of interest.
