# Supplementary material for: Enhanced Energy Conversion of Z907-Based Solar Cells by Cucurbit[7]uril Macrocycles
Source: Front Chem. 2019 Aug 8;7:561. doi: 10.3389/fchem.2019.00561 (PMC6694293; doi:10.3389/fchem.2019.00561)
Supplement: Supplementary file 1 [file Data_Sheet_1.pdf]

SUPPORTING INFORMATION

Enhanced Energy Conversion of Z907-Based Solar Cells by Cucurbit[7]uril Macrocycles

Hmoud Al-Dmour,[‡] Reem H. Alzard,[§] Hamda Alblooshi,[§] Khaula Alhosani,[§] Shaqra AlMadhoob,[§] and Na'il Saleh*[§]

[‡]*Department of Physics, Faculty of Science, Mu'tah University, Mu'tah, 61710, Jordan.*

[§]*Chemistry Department, College of Science, United Arab Emirates University, P.O. Box 15551, Al Ain, United Arab Emirates*

TABLE OF CONTENTS

Part 1: Titrations……………………………………………………………..….…………S3

Titration by UV−vis absorption method. ………………………………….……….…S3

Titration by NMR method. ………………………………………………..……..…S5

Part 2: Excitation spectra in solution ………………………..…………….……..…S4

Excitation spectra…………………... ……………………………….……….…S4

Excitation-dependence emission spectra …..……….…………………..……..…S4

Part 3: FTIR Spectra …………………………………………….………...……..… S6

Part 4: TRPL measurements …………………..……………………….…………S7

Emission decays in solution ….……......…………………………….……….…S7

Emission decays on electrodes ……..… …..………….…………..……..…S8

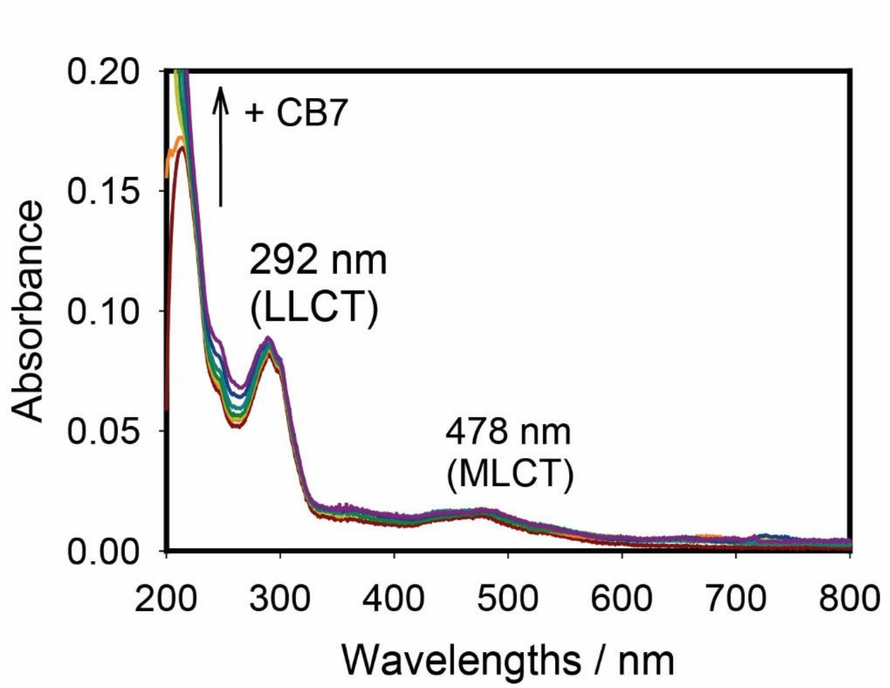

Figure S1. UV–vis absorption bands of Z907 (30 μM) in acetonitrile and water (1:9 v/v) in the absence and presence of 0−50 equivalents CB7. No changes were observed.

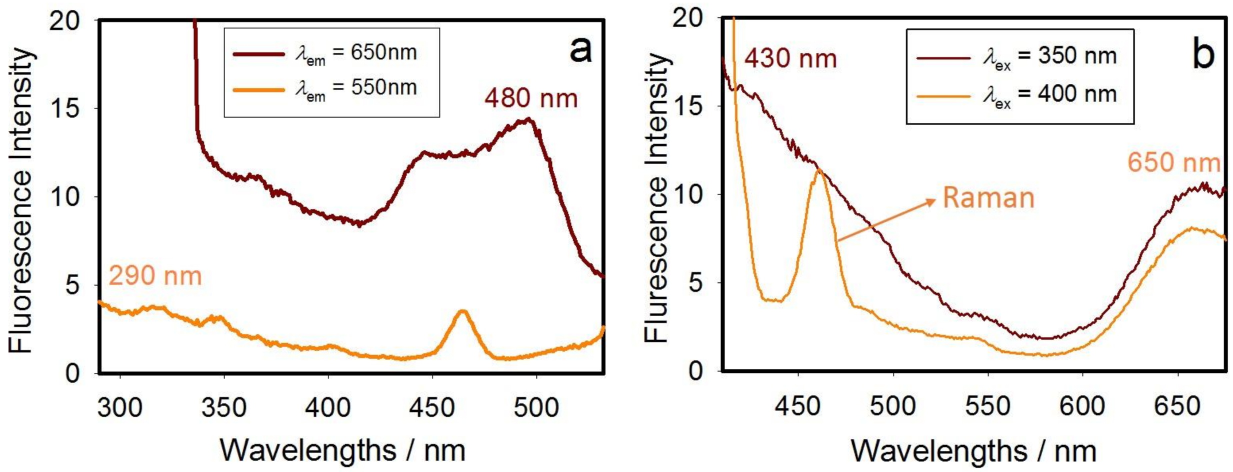

Figure S2. Excitation spectra (a) monitored at 550 and 650 nm of Z907 (30 µM) in in acetonitrile and water (1:9 v/v). And excitation-dependence emission spectra (b) at 350 and 400 nm of Z907 (30 µM) in in acetonitrile and water (1:9 v/v).

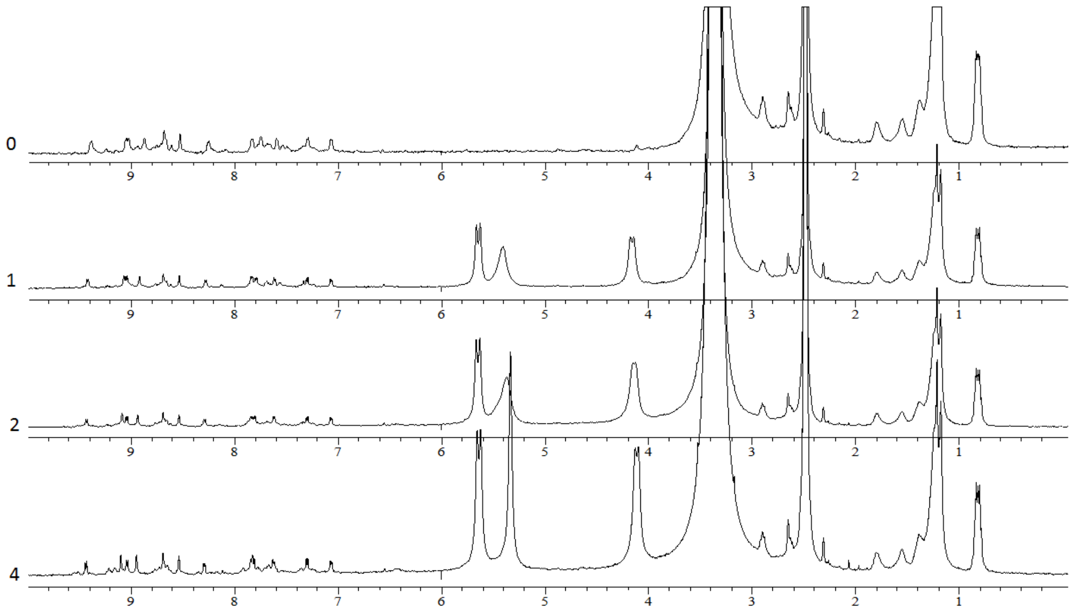

Figure S3. ^{1}H-NMR titration of Z907 (1.0 mM) in DMSO-d_6 in the absence and presence of 0−4 equivalents CB7. No changes were observed.

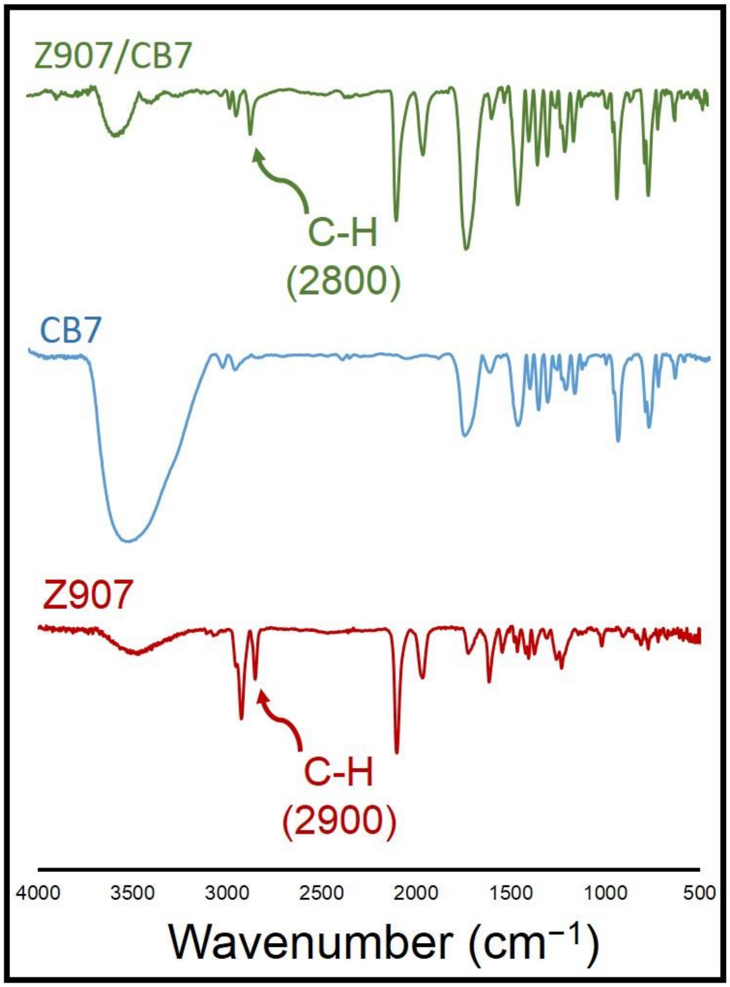

Figure S4. FTIR spectra of Z907, CB7 and their solid complex in KBr desk.

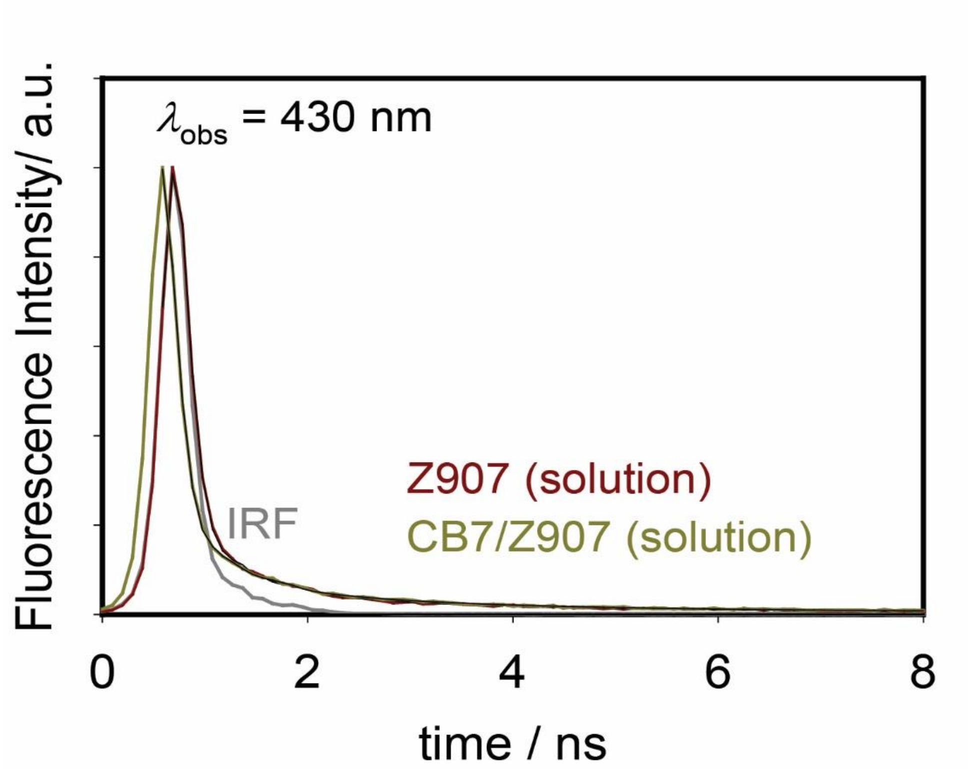

Figure S5. Emission decays monitored at 430 nm for Z907 (30 μM) in acetonitrile/water (1:9 v/v, pH 7) and inside CB7 (10 equivalents) upon excitation at 375 nm and room temperature.

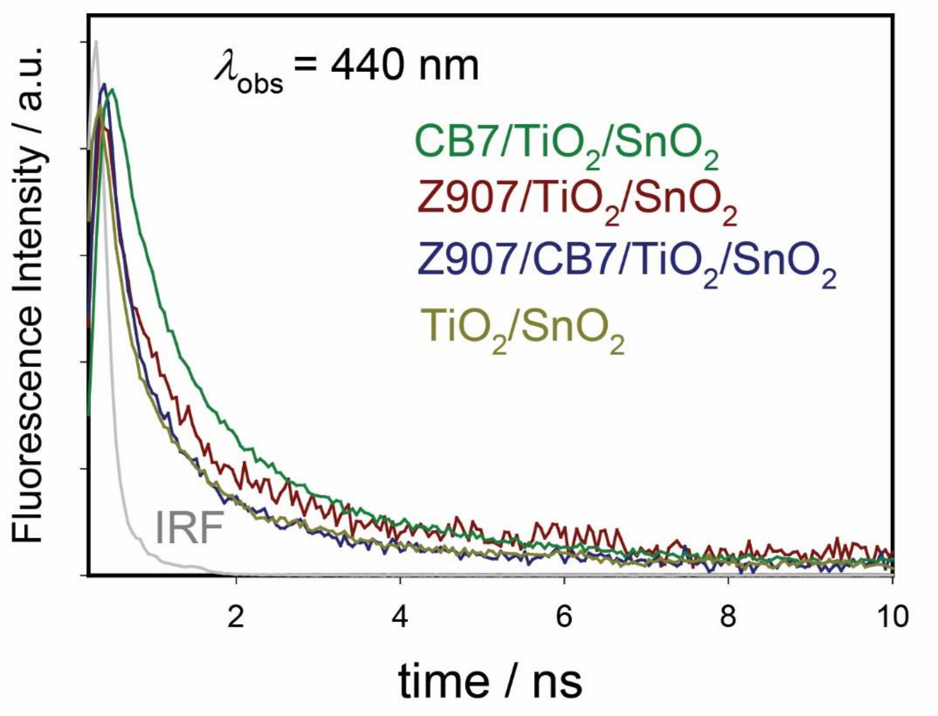

Figure S6. Emission decays at 440 nm of Z907/TiO_2/SnO_2 and Z907/CB7/TiO_2/SnO_2 electrodes along with CB7-coated and uncoated TiO_2/SnO_2 electrodes.
